# The causal associations between 25(OH) vitamin D levels and ectopic pregnancy: A 2-sample Mendelian randomization study

**DOI:** 10.1097/MD.0000000000050657

**Published:** 2026-09-18

**Authors:** Pei-Jin Zhang, Shao Bo, Shuang Liang, Hong-Yuan Zhang, Xiao-Dong Fan

**Affiliations:** a Department of Gynecology, Tianjin Central Hospital of Obstetrics and Gynecology, Tianjin, China; b Department of General Surgery, Tianjin Medical University General Hospital, Tianjin, China.

**Keywords:** causal effect, ectopic pregnancy, Mendelian randomization, vitamin D

## Abstract

Previous research has indicated a potential link between 25-hydroxyvitamin D [25(OH)D] and ectopic pregnancy (EP), but the underlying causal relationship remains unclear. We performed a 2-sample Mendelian randomization (MR) study to investigate the causal association between circulating 25(OH)D and ectopic pregnancy (EP). The primary analysis used summary statistics from a 25(OH)D GWAS of 4,96,946 European-ancestry participants and FinnGen summary statistics for EP (3111 cases and 89,340 controls). To assess whether the finding was robust to the choice of 25(OH)D GWAS, we repeated the MR analysis using a separate 25(OH)D GWAS (n = 4,41,291 European-ancestry participants) as a replication analysis; it was not used solely as a within-analysis sensitivity test. The primary and replication analyses used inverse-variance weighting (IVW) as the main method, complemented by weighted mode, simple mode, weighted median, and MR-Egger analyses. Heterogeneity and pleiotropy assessments, leave-one-out analyses, and other sensitivity analyses were conducted within each MR analysis. IVW estimates showed that increased serum 25(OH)D levels promoted EP (OR = 1.323, 95% CI = 1.024–1.710, *P* = .032). The replication analysis also revealed similar trends (OR = 1.383, 95% CI = 1.041–1.836, *P* = .025). The primary and replicated analyses were combined in a meta-analysis, and the result showed a significant causal influence (OR = 1.350, 95% CI = 1.116–1.632, *P* = .002). Sensitivity analysis confirmed the robustness of the MR estimations. In this MR study of European-ancestry participants, genetically predicted higher circulating 25(OH)D levels were associated with a higher risk of EP. These findings do not establish serum 25(OH)D as a diagnostic marker for EP. Rather, if confirmed in further clinical studies, elevated vitamin D may be considered a potential associated risk factor only in the context of established clinical signs and symptoms of EP.

## 1. Introduction

The term “ectopic pregnancy” (EP) describes an embryo implanted outside of the uterus,^[1]^ which is considered one of the main causes of mortality (10%) and morbidity (1–2%) causes among pregnant women in the first trimester of pregnancy.^[2]^ Abdominal pain and vaginal bleeding are some common symptoms of ectopic pregnancy.^[3]^ Over 90% of instances of EP occur in the fallopian tube, while it can also develop in the cervix, ovary, myometrium, and abdominal cavity.^[4]^ A number of risk factors have been associated with EP,^[5]^ these include smoking, alcohol consumption, tubal surgery or tubal injury, history of EP, IUD use, prior pelvic infection, and pregnancy conceived by assisted reproduction. In an effort to effectively manage the symptoms and prevent it from occurring again, more focus has been placed on the rising risk factors of EP.

Vitamin D (Vit D) has attracted increasing attention in female reproductive biology because Vit D receptor expression and Vit D-related signaling pathways have been identified in the endometrium, uterus, ovaries, placenta, and fallopian tube.^[6,7]^ A pleiotropic lipid-soluble vitamin, Vit D not only regulates phosphorus and calcium metabolism but also plays an important role in regulating cell differentiation, proliferation, and apoptosis, while also having immune-modulating properties.^[8]^ The Vit D target organs are present in many parts of the body, and different forms of Vit D bind to different receptors to carry out various tasks.^[9]^ According to reports, endometriosis,^[10]^ uterine fibroids,^[11]^ and polycystic ovarian syndrome^[12]^ are linked to Vit D deficiency. Vahedpoor et al found that vitamin D supplements had effects on intraepithelial lesion of the uterine cervix regression.^[13]^ The most widely utilized biomarker of Vit D levels has been 25-hydroxyvitamin D, or 25(OH)D, the most stable form in circulation.^[14]^ Given its relatively stable circulating concentration, 25(OH)D is widely used to assess vitamin D exposure in epidemiological studies. In recent years, limited reports indicated that although 25(OH)D has a protective impact against ectopic pregnancy, there is still uncertainty about the association between 25(OH)D levels and the risk of ectopic pregnancy, and further research is required to clarify this.^[15,16]^

Mendelian randomization (MR) is a method used to evaluate the relationship between a risk factor and a disease by utilizing genetic variants as instrumental variables (IVs) to represent the risk factor of interest. These genetic variants are often single-nucleotide polymorphisms (SNPs).^[17]^ MR may evaluate the impact of lifetime exposure on the risk of an outcome since genetic variation is inherited and stays relatively constant,^[18]^ as parental alleles are randomly passed on to their offspring.^[19]^ Many of the drawbacks of conventional observational research, such as sample size restrictions, reverse causality, and residual confounding, are usually circumvented using MR. MR can provide a strong estimate between an outcome of interest and a risk factor, strengthening the inference of causality.^[20]^ To date, the causal association between circulating 25(OH)D concentrations and EP has not been investigated using an MR approach. Therefore, we conducted a 2-sample MR study to evaluate whether genetically predicted circulating 25(OH)D levels are causally associated with the risk of EP.

## 2. Materials and methods

### 2.1. Study design

**Figure 1.**
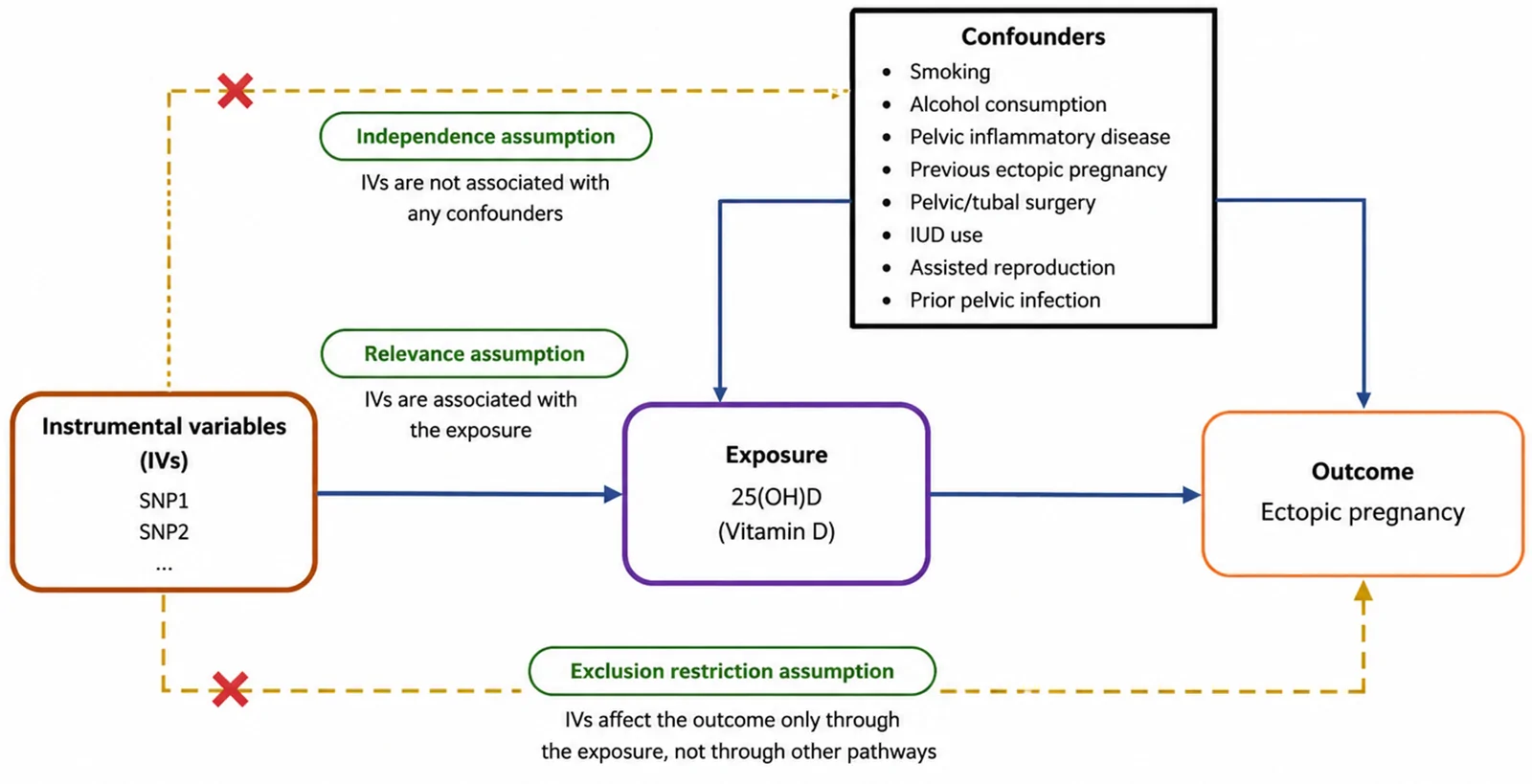
Directed acyclic graph illustrating the Mendelian randomization framework. Genetic variants associated with circulating 25(OH)D levels were used as instrumental variables. Valid causal inference requires: (1) relevance, whereby genetic variants are strongly associated with circulating 25(OH)D levels; (2) independence, whereby genetic variants are not associated with confounders of the association between 25(OH)D and ectopic pregnancy; and (3) exclusion restriction, whereby genetic variants affect ectopic pregnancy only through circulating 25(OH)D levels rather than through alternative pathways. In this conceptual DAG, the confounder node represents potential confounders and clinically recognized EP risk factors, including smoking, alcohol consumption, pelvic inflammatory disease or other pelvic/tubal infection, previous ectopic pregnancy, prior tubal or pelvic surgery or injury, intrauterine device use, conception by assisted reproductive technology, and relevant metabolic and reproductive/gynecological traits. The figure does not imply that all clinical risk factors were available as GWAS phenotypes; PhenoScanner screening was performed only for phenotypes represented in the database. 25(OH)D = 25-hydroxyvitamin D, DAG = directed acyclic graph, EP = ectopic pregnancy, IUD = intrauterine device, IV = instrumental variable, SNP = single-nucleotide polymorphism.

This study was designed as a 2-sample MR study using publicly available genome-wide association study (GWAS) summary statistics to evaluate whether genetically predicted 25(OH)D levels are associated with the risk of ectopic pregnancy. MR analysis relies on 3 core assumptions. First, the relevance assumption requires that the selected genetic variants are strongly associated with circulating 25(OH)D levels. Second, the independence assumption requires that the genetic variants are not associated with confounders of the association between 25(OH)D and ectopic pregnancy. Third, the exclusion restriction assumption requires that the genetic variants influence ectopic pregnancy only through circulating 25(OH)D levels and not through alternative pathways.^[21]^ Figure 1 presents the directed acyclic graph (DAG) illustrating these assumptions.

To assess the relevance assumption, single-nucleotide polymorphisms (SNPs) associated with 25(OH)D at genome-wide significance (*P* < 5 × 10^−8^) were selected, pruned for linkage disequilibrium, and evaluated using *F*-statistics; an *F*-statistic > 10 was considered adequate evidence against weak-instrument bias. To reduce potential violation of the independence assumption, the exposure and outcome GWAS datasets were restricted to participants of European ancestry. In addition, all candidate index SNPs were queried in the PhenoScanner GWAS database at a genome-wide significance threshold (*P* < 5 × 10^−8^). We prespecified 3 confounder domains relevant to EP: lifestyle factors, including smoking initiation, current/ever smoking, cigarettes per day, alcohol consumption, and alcohol-use phenotypes; metabolic factors, including body mass index, obesity, waist-hip measures, type 2 diabetes, fasting glucose, fasting insulin, and lipid traits; and reproductive/gynecological factors, including endometriosis, polycystic ovary syndrome, uterine fibroids, female infertility, pelvic inflammatory disease or tubal disease, sexually transmitted infection-related phenotypes, and reproductive timing or fertility-treatment traits, where available in the database. SNPs with reported associations with any prespecified potential confounder were excluded. Because GWAS coverage was limited for several clinical EP risk factors, absence of a cataloged association was not interpreted as proof that an instrument was free from confounding. To evaluate potential violation of the exclusion restriction assumption, we performed the MR-Egger intercept test and the MR-PRESSO global test to assess horizontal pleiotropy and identify outlying variants. Cochran *Q* test, leave-one-out analysis, and complementary MR methods were used to assess heterogeneity and the robustness of the causal estimates. Because the independence and exclusion restriction assumptions cannot be directly proven using summary-level data, these analyses were interpreted as tests for evidence of major violations rather than definitive verification.

In the DAG, the confounder node represented potential confounders and clinically recognized EP risk factors. In addition to smoking and alcohol consumption, these included pelvic inflammatory disease or other pelvic/tubal infection, previous ectopic pregnancy, prior tubal or pelvic surgery or injury, intrauterine device use, and conception by assisted reproductive technology, as well as relevant metabolic and reproductive/gynecological factors. These factors were considered in the PhenoScanner screen when corresponding GWAS phenotypes were available.

To reduce the possibility that the causal estimate was driven by the selection of a single 25(OH)D GWAS or its corresponding genetic instruments, we performed both a primary MR analysis and a replication MR analysis. The primary analysis used the main 25(OH)D GWAS summary statistics, whereas the replication analysis used a separate 25(OH)D GWAS from the IEU GWAS resource (GWAS ID: ieu-b-4808). In both analyses, the FinnGen ectopic pregnancy GWAS served as the outcome dataset (3111 cases and 89,340 controls). Thus, the second 25(OH)D GWAS was used to replicate the main finding using an alternative set of SNP-25(OH)D estimates, not as a substitute for the within-analysis sensitivity analyses. Sensitivity analyses, including MR-Egger, MR-PRESSO, heterogeneity testing, and leave-one-out analysis, were performed separately within each analysis. All study participants were of European ancestry to reduce population stratification. SNP selection and sensitivity analyses were conducted according to established MR guidelines.^[22]^

In conducting our analysis, we utilized publicly available GWAS summary statistics. Given that our study was predicated exclusively on these preexisting, anonymized data sets, there was no requirement for the collection of fresh data or the procurement of additional ethical clearance.

### 2.2. Genetic association dataset for vitamin D

The most widely utilized biomarker of vitamin D status has been 25(OH)D, the most stable form in circulation.^[14]^ Using 4,96,946 participants of European ancestry from a genome-wide association study (GWAS) summary dataset, we selected SNPs associated with serum 25(OH)D levels. The initial GWAS identified 103 SNPs (Table S1, Supplemental Digital Content 1). To assess potential confounding, we performed a structured PhenoScanner screening of the candidate SNPs, using the GWAS catalog and a significance threshold of *P* < 5 × 10^−8^. The screen included the prespecified lifestyle, metabolic, and reproductive/gynecological trait categories described above. In particular, we evaluated smoking- and alcohol-related phenotypes; metabolic phenotypes, including adiposity, glycemic, and lipid traits; and reproductive/gynecological and clinical EP-risk phenotypes, including endometriosis, polycystic ovary syndrome, uterine fibroids, infertility, pelvic inflammatory disease or other pelvic/tubal infection, previous ectopic pregnancy, prior tubal or pelvic surgery or injury, intrauterine device use, and reproductive timing or assisted-reproduction/fertility-treatment traits, where available in the database. SNPs associated with a potential confounder were excluded before MR estimation. This phenotype screen was intended to reduce, but could not eliminate, the possibility of pleiotropy through unmeasured or incompletely represented clinical EP risk factors.^[23-26]^

Independent SNPs associated with 25(OH)D at the genome-wide significance level (*P* < 5 × 10^−8^) were selected using a 10,000 kb clumping window, a minor allele frequency > 0.01, and an LD threshold of *r*^2^ < 0.001. LD estimates were based on European-ancestry samples from the 1000 Genomes Project. To ensure transparent reporting of instrument selection, we recorded the number of variants retained after LD pruning, extraction in the FinnGen outcome dataset, harmonization, and PhenoScanner screening. Instrument strength was assessed using the *F*-statistic, with *F* > 10 considered indicative of a sufficiently strong instrument. During harmonization, SNPs with ambiguous palindromic alleles or incompatible effect alleles were excluded to ensure alignment of exposure and outcome effects.

For the replication analysis, we used a separate GWAS summary dataset for circulating 25(OH)D from the IEU GWAS resource (GWAS ID: ieu-b-4808; n = 4,41,291 participants of European ancestry; Table S1, Supplemental Digital Content 1). This dataset was selected to evaluate whether the primary MR finding could be reproduced when SNP-25(OH)D associations and instrumental variables were derived from an alternative exposure GWAS. It was therefore used as a replication dataset rather than solely for sensitivity analysis. SNPs for the replication analysis were selected using the same criteria as in the primary analysis: genome-wide significance (*P* < 5 × 10^−8^), minor allele frequency > 0.01, a 10,000 kb clumping window, and LD *r*^2^ < 0.001. The numbers of SNPs retained after LD pruning and after harmonization were recorded using the same workflow as for the primary analysis.

### 2.3. Genetic instrumental variables for ectopic pregnancy

Ectopic pregnancy outcome data were obtained from the FinnGen R5 release via the IEU OpenGWAS resource (GWAS ID: finn-b-O15_PREG_ECTOP), comprising 3111 cases and 89,340 controls (Table S1, Supplemental Digital Content 1). The FinnGen ectopic-pregnancy endpoint was restricted to females and defined from registry-recorded diagnoses in the Hospital Discharge Register and the Cause of Death Registry, using ICD-10 code O00, ICD-9 code 633, and ICD-8 code 631. The publicly available endpoint documentation does not report individual-level confirmation by ultrasound, surgery, pathology, or chart review, nor does it provide an endpoint-specific formal validation metric (e.g., positive predictive value) for this coding algorithm. Therefore, the outcome in the present study should be interpreted as registry-defined ectopic pregnancy rather than individually clinically adjudicated ectopic pregnancy. Although FinnGen endpoints are clinically curated and use harmonized diagnostic codes across ICD revisions, some diagnostic misclassification cannot be excluded. The same FinnGen outcome dataset was used in both the primary and replication MR analyses. Selecting an outcome GWAS distinct from the vitamin D exposure datasets was intended to reduce the potential for bias due to sample overlap.^[18]^

## 3. Statistical analysis

The primary analysis used random-effects inverse-variance weighting (IVW) to estimate odds ratios (ORs) and 95% confidence intervals (CIs) for the association between genetically predicted circulating 25(OH)D levels and ectopic pregnancy.^[27,28]^ Weighted median, weighted mode, simple mode, and MR-Egger analyses were performed as complementary approaches.^[29-31]^ Agreement across these methods was considered supportive of the robustness of the causal estimate under different assumptions regarding instrument validity. Leave-one-out analysis was performed using IVW to evaluate whether the estimate was driven by a single SNP.^[19]^ Cochran *Q* statistic was used to assess heterogeneity among SNP-specific estimates.^[32]^ The MR-Egger intercept test and MR-PRESSO global test were used to assess potential horizontal pleiotropy and identify outlying variants, thereby evaluating potential violations of the exclusion restriction assumption. A 2-sided *P* value < .05 was considered statistically significant.^[33]^ All analyses were conducted in R version 4.3.0 (R Foundation for Statistical Computing, Vienna, Austria) using the Two Sample MR package (version 0.5.6) and MR-PRESSO package (version 1.0). All analyses were conducted in R version 4.3.0. Instrument SNP extraction, outcome SNP extraction, LD clumping, and allele harmonization were performed using the extract_instruments(), extract_outcome_data(), clump_data(), and harmonise_data() functions in the TwoSampleMR package (version 0.5.6). The MR-PRESSO package (version 1.0) was used for MR-PRESSO global and outlier analyses.

Cochran *Q* statistics were calculated separately for the IVW and MR-Egger models. A 2-sided *P* value < .05 for Cochran *Q* was prespecified as evidence of statistically significant heterogeneity, whereas *P* ≥ .05 was interpreted as no statistically significant evidence of heterogeneity. Because a nonsignificant *Q* test does not establish complete homogeneity, the *Q*-test results were interpreted together with the random-effects IVW estimates and the complementary sensitivity analyses.

## 4. Results

For the primary analysis, 103 genome-wide significant candidate SNPs associated with serum 25(OH)D were initially identified, and all 103 SNPs were retained after LD pruning (*r*^2^ < 0.001 within a 10,000-kb window). All 103 SNPs were available in the FinnGen ectopic pregnancy outcome dataset. Following harmonization, 100 SNPs were retained: 2 palindromic SNPs (rs57601828 and rs7955128) and 1 SNP with incompatible alleles were excluded. All 100 harmonized SNPs had *F*-statistics >30.45, indicating adequate instrument strength and a low likelihood of weak-instrument bias.^[26]^ In the PhenoScanner screen, 4 harmonized SNPs with reported associations with prespecified potential confounder phenotypes were excluded: rs1260326, rs3114045, rs7652808, and rs13108245. The reported associations involved lifestyle/behavioral and/or metabolic domains; rs1260326, in particular, has documented associations with metabolic traits. Reproductive and gynecological phenotypes were also evaluated where available in the database. No additional retained SNP was excluded solely on the basis of a cataloged reproductive/gynecological association at the prespecified significance threshold. Therefore, 96 SNPs were retained as instrumental variables for the primary MR analysis (Table S2, Supplemental Digital Content 2). These findings reduce, but do not exclude, the possibility of residual pleiotropy through incompletely measured reproductive risk factors.

Initially, the work tested for causal effects using the IVW approach. We discovered that the risk of EP rose with an increase in serum 25(OH)D levels [odds ratio (OR) = 1.323, 95% CI: 1.024–1.710, *P* = .032]. These findings demonstrate a direct correlation between serum 25(OH)D levels and EP risk in the European populace. Next, in order to investigate and confirm the causal association between serum 25(OH)D levels and EP, we used 4 alternative models. The IVW direction was supported by the findings of the weighted model, simple model, and MR-Egger regression (Table 1).

**Table 1:** Associations between genetically predicted 25(OH)D and risk of ectopic pregnancy.

Exposure	Outcome	Nsnp	Methods	Beta	SE	OR (95% CI)	*P*
Serum 25(OH)D	Ectopic pregnancy	96	MR Egger	0.027	0.201	1.028 (0.693–1.524)	.893
			Weighted median	0.142	0.224	1.152 (0.743–1.788)	.527
			Inverse variance weighted	0.28	0.131	1.323 (1.024–1.710)	.032
			Simple mode	0.381	0.438	1.464 (0.621–3.452)	.386
			Weighted mode	0.094	0.189	1.099 (0.758–1.592)	.62
Circulating 25(OH)D	Ectopic pregnancy	166	MR Egger	0.181	0.237	1.199 (0.753–1.909)	.446
			Weighted median	0.147	0.259	1.158 (0.698–1.922)	.571
			Inverse variance weighted	0.324	0.145	1.383 (1.041–1.836)	.025
			Simple mode	0.382	0.616	1.465 (0.438–4.900)	.536
			Weighted mode	0.183	0.229	1.200 (0.766–1.881)	.427

CI = confidence interval, OR = odds ratio, SE = standard error, SNP = single-nucleotide polymorphism.

*P* < .05 was considered statistically significant.

**Figure 2.**
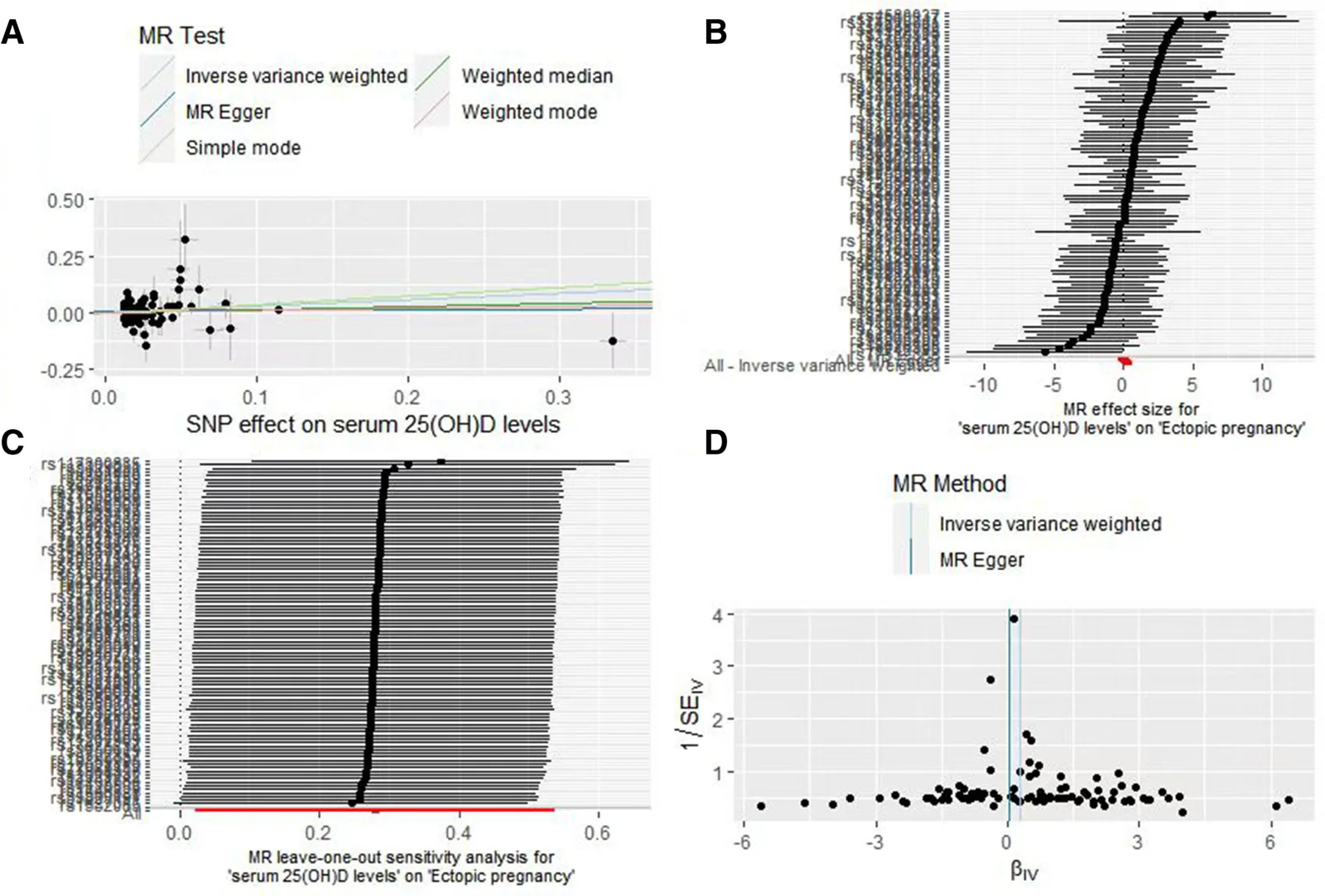
Scatter plot (A), forest plot (B), leave-one-out analysis (C), and funnel plot (D) for the primary MR analysis of circulating 25(OH)D and ectopic pregnancy, using the primary 25(OH)D GWAS and the FinnGen ectopic pregnancy outcome dataset. 25(OH)D = 25-hydroxyvitamin D, GWAS = genome-wide association study, MR = Mendelian randomization, SE = standard error, SNP = single-nucleotide polymorphism.

The consistency of results across the 5 MR methods supported the robustness of the association between serum 25(OH)D levels and ectopic pregnancy (Fig. 2A). The forest and funnel plots are shown in Figure 2B, D, respectively. Leave-one-out analysis showed that removal of any single SNP did not materially alter the overall causal estimate (Fig. 2C). In addition, Cochran *Q* test showed no evidence of statistically significant heterogeneity in either IVW or MR-Egger analyses (both *P* > .05; Table 2). According to the prespecified criterion (*P* < .05), these findings indicated no statistically significant evidence of heterogeneity. After 2000 MR-PRESSO simulations, no outliers were identified, and no evidence of horizontal pleiotropy was observed (*P* > .05; Table 2). Together with the nonsignificant MR-Egger intercept, these findings provided no evidence of a major violation of the exclusion restriction assumption. These sensitivity analyses support, but do not definitively prove, the validity of the MR assumptions.

**Table 2:** Sensitivity analysis of 25(OH)D genetic IVs in GWAS for ectopic pregnancy.

Exposure		Outcome	Heterogeneity test						Pleiotropy test				
			MR_Egger			IVW			MR_Egger				PRESSO
			*Q*	*Q*_df	*Q*_pval	*Q*	*Q*_df	*Q*_pval	Intercept	SE	*P*		*P*
Serum 25(OH)D	Ectopic pregnancy		95.327	94	0.442	98.073	95	0.394	0.009	0.005		.103	.44
Circulating 25(OH)D	Ectopic pregnancy		168.108	164	0.397	168.7	165	0.406	0.003	0.004		.449	.181

25(OH)D = 25-hydroxyvitamin D, GWAS = genome-wide association study, IV = instrumental variable, IVW = inverse-variance weighted, MR = Mendelian randomization, MR-PRESSO = Mendelian Randomization Pleiotropy RESidual Sum and Outlier, SE = standard error.

**Figure 3.**
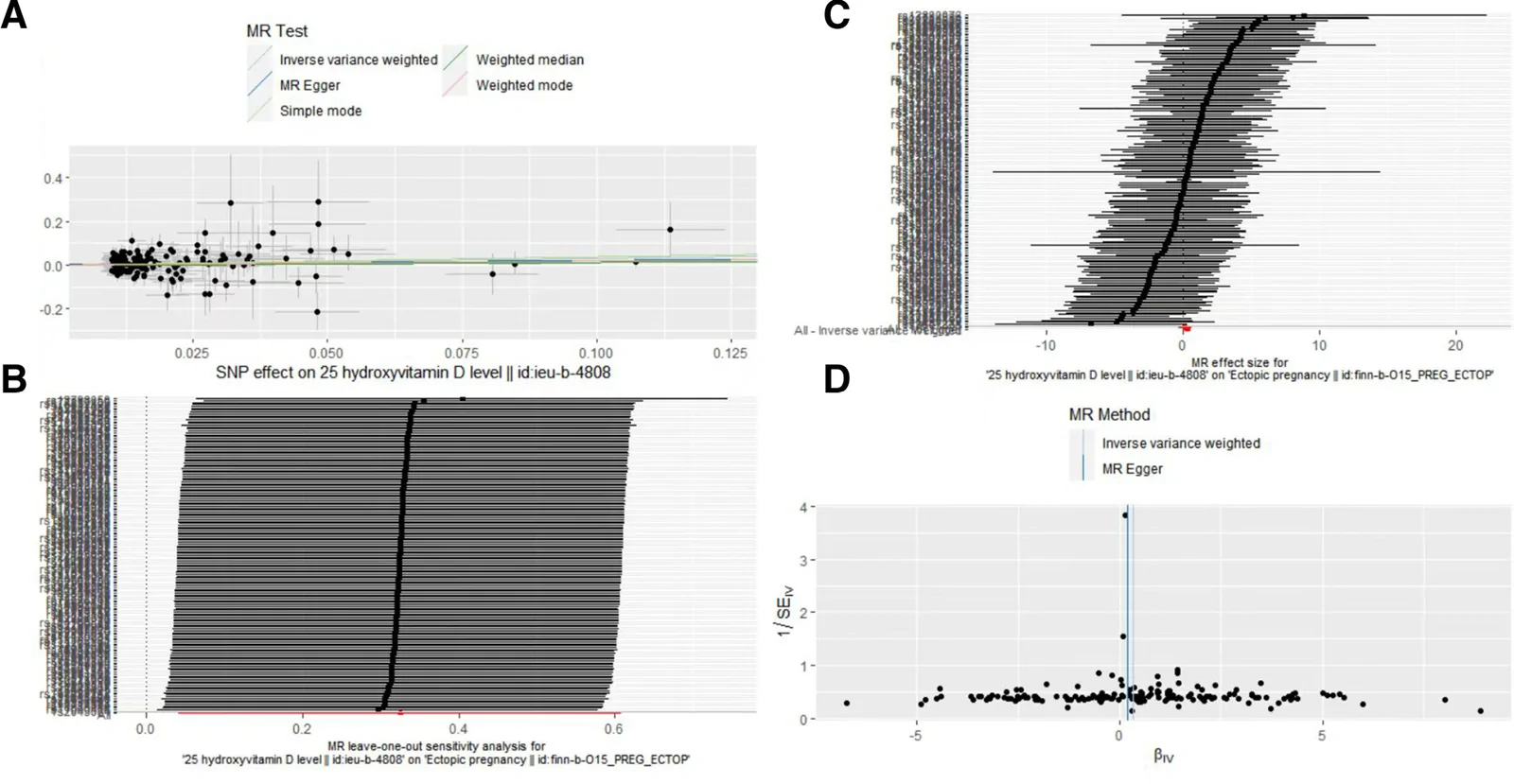
Scatter plot (A), forest plot (B), leave-one-out analysis (C), and funnel plot (D) for the replication MR analysis of circulating 25(OH)D and ectopic pregnancy, using the alternative 25(OH)D GWAS (ieu-b-4808) and the FinnGen ectopic pregnancy outcome dataset. 25(OH)D = 25-hydroxyvitamin D, GWAS = genome-wide association study, MR = Mendelian randomization, SE = standard error, SNP = single-nucleotide polymorphism.

**Figure 4.**
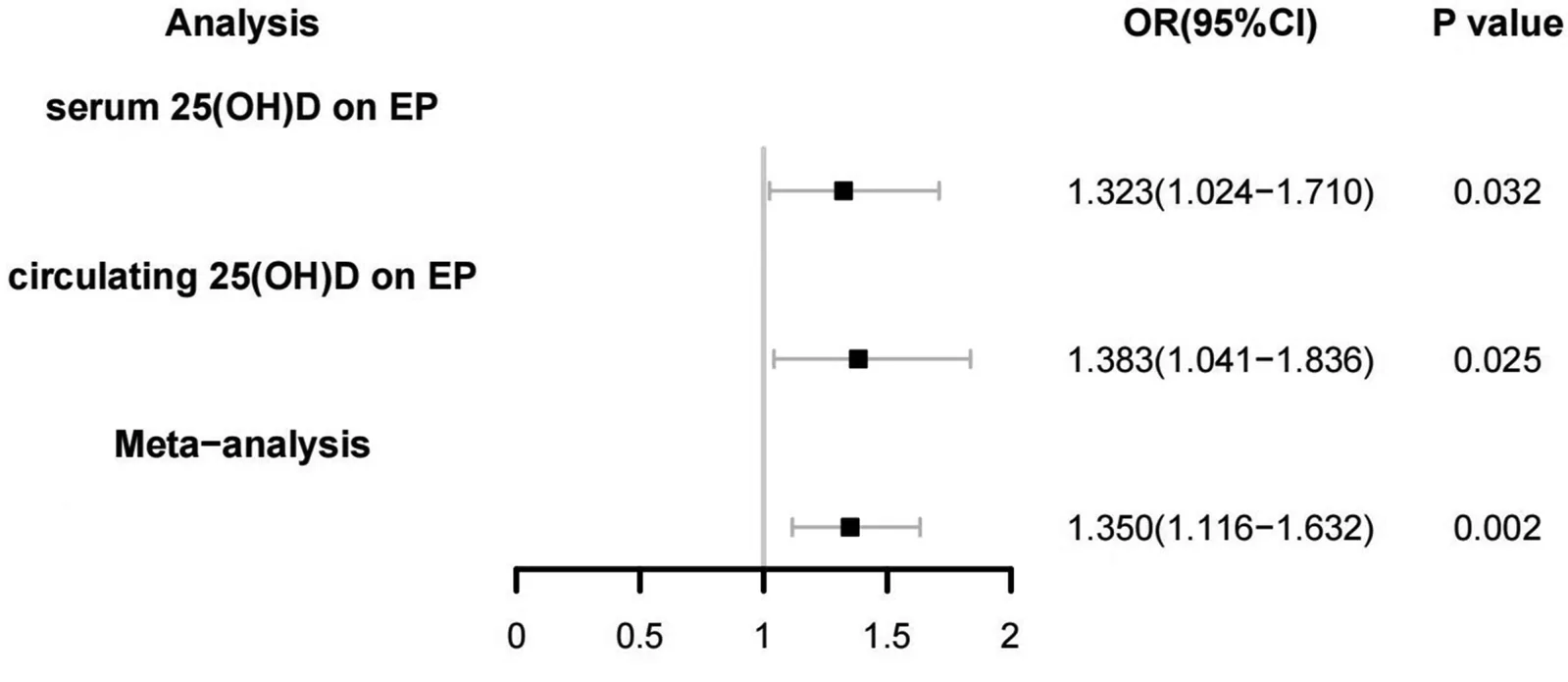
Meta-analysis of the primary and replication MR estimates for the effect of genetically predicted circulating 25(OH)D levels on ectopic pregnancy. 25(OH)D = 25-hydroxyvitamin D, CI = confidence interval, EP = ectopic pregnancy, OR = odds ratio, SNP = single-nucleotide polymorphism.

Replication analysis using the separate 25(OH)D GWAS (ieu-b-4808; n = 4,41,291) was performed to examine whether the primary result was reproducible when the SNP-25(OH)D associations were derived from an alternative exposure dataset; it was not conducted merely as a sensitivity analysis. Using the same FinnGen EP outcome dataset, the replication IVW estimate showed that higher genetically predicted circulating 25(OH)D levels were associated with an increased risk of EP (OR = 1.383, 95% CI: 1.041–1.836, *P* = .025). Results from weighted median, MR-Egger, simple mode, and weighted mode analyses showed a consistent direction of effect (Table 2; Fig. 3). No evidence of significant heterogeneity or horizontal pleiotropy was detected (both *P* > .05; Table 2). Additional analyses showed no evidence that the replication instrument set was associated with smoking or alcohol use (Table S1, Supplemental Digital Content 1; Table 3). Meta-analysis of the primary and replication IVW estimates showed a statistically significant association between higher genetically predicted 25(OH)D levels and EP risk (OR = 1.350, 95% CI: 1.116–1.632, *P* = .002; Fig. 4).

**Table 3:** The IVW estimates of circulating 25(OH)D genetic IVs on the EP potential risk factors.

Exposure	Outcome	Beta	SE	OR (95% CI)	*P*
Circulating 25(OH)D	Smoking	−0.002	0.01	0.998 (0.977–1.018)	.82
	Alcohol consumption	−0.019	0.014	0.982 (0.956–1.008)	.175

25(OH)D = 25-hydroxyvitamin D, CI = confidence interval, IV = instrumental variable, IVW = inverse-variance weighted, OR = odds ratio, SE = standard error.

## 5. Discussion

Vitamin D, being a naturally occurring active substance, has various roles and is broadly disseminated in vivo through its receptors. Additionally, vitamin D receptors were found to be present in the uterus, endometrium, ovaries, and placenta of females. *In* vivo studies revealed that blockage of vitamin D receptors in rats leads to uterine hyperplasia.^[34]^ It has been shown that atypical expression of vitamin D metabolizing and signaling pathways by binding to vitamin D receptors on the surface of the fallopian tube could result in tubal gestation.^[35]^ Through a variety of signaling pathways, vitamin D primarily regulates the levels of inflammatory factors, including cytokines, interleukins, and prostaglandins, which play critical roles in the processes of endometrial receptivity and embryo implantation.^[36]^ Additionally, a study discovered that vitamin D can suppress the expression of progesterone and estrogen receptors, which in turn reduces the effectiveness of progesterone and estrogen in endocrine functions^[37]^ These data collectively imply that vitamin D is crucial for the development of EP.

Abedini et al^[15^ reported that 94% of women who experienced an ectopic pregnancy had inadequate blood vitamin D levels in their serum. Sahhaf et al^[16]^ reported that 60.66% of women with ectopic pregnancy had vitamin D deficiency and 21.33% of patients had vitamin D insufficiency. According to these randomized clinical trials (RCTs), pregnant women in good health had much higher vitamin D levels than pregnant women experiencing an ectopic pregnancy.

On the other hand, our findings showed that the primary IVW estimate demonstrated a causal relationship between elevated 25(OH)D levels and an elevated risk of EP (OR = 1.350, 95% CI: 1.116–1.632, *P* = .002), which is opposite to the results of the former RCT studies. We speculate it may probably be related to the following factors. First of all, in clinical trials, confounding variables and heterogeneity – such as varied research designs and populations – will always interfere. Mendel’s law of random assignment and SNPs are used in MR research as instrumental variables to reduce the impact of confounding variables and guarantee that study subjects are homogeneous, which would strengthen the findings. Chronically elevated vitamin D levels are the cause of the result, which is reproduced by a lifetime exposure in the MR study. In addition, we conducted a meta-analysis and repeated the analysis using a different independent GWAS dataset. To prevent violations, a number of sensitivity studies were carried out in addition to identifying potential risk variables. As a consequence, our findings strongly suggested that 25(OH)D causes EP. Second, we chose our data from a summary collection of GWAS. The mean vitamin D level of Western women in Vander Meer’s study was 3 times higher than that of Non-Western women.^[38]^ Increasing vitamin D intake on a high-level basis may increase bio-toxicity. Thirdly, in a study by Ozkan et al,^[39]^ following embryo transfer, women with greater serum vitamin D levels had a markedly increased chance of becoming pregnant. Therefore, we speculate that vitamin D can increase the overall chance of pregnancy and increase the risk of ectopic pregnancy. Last but not least, the body uses vitamin D through a complicated system. Additionally, through vitamin D levels, certain genetic variations have been shown to influence the risk of EP. While there are various ways in which genetic variables might impact vitamin D, other genes unrelated to vitamin D and the signaling pathways associated with them may contribute to the development of EP. Consequently, only a portion of the risk of EP can be explained by variations linked to vitamin D; further analysis of other signaling pathways is required to fully comprehend the remaining risk factors for EP.

Our analysis has several key strengths; first, we were able to investigate more trustworthy causal associations because the chosen GWAS datasets had a sizable sample size with millions of SNPs identified. Most significantly, we performed many analyses to support the robustness of the estimation and a replicate analysis to guarantee the stability of the results.

Secondly, the GWAS datasets for ectopic pregnancy and 25-hydroxyvitamin D genetic instrumental variables were sourced from European individuals, minimizing the impact of population stratification. Additionally, these datasets had large sample sizes with millions of SNPs, enhancing the reliability of the causal association analysis. Most importantly, to ensure the stability of our findings, we performed a replication analysis and applied several additional methods to further support the robustness of the estimation. In this work, we establish a solid foundation for future investigations into the potential role of vitamin D as a causal risk factor for EP, which could have significant public health ramifications.

There are a few study limitations, nevertheless, that should also be taken into account. First, more research is required to generalize our findings to other populations. Second, more research is required to determine the exact processes by which genetically elevated 25(OH)D raises the risk of EP in the European population. Third, because we were limited to using summary-level information, stratification analysis was not allowed. It will be important to design future randomized clinical trials to thoroughly examine the possible impact of vitamin D or its combination with other medications on the prevention and treatment of EP.

There are several limitations that should be considered. First, additional studies are required to determine whether our findings can be generalized to populations of non-European ancestry. Second, further work is needed to identify the biological mechanisms through which genetically predicted 25(OH)D levels may influence the risk of EP. Third, because the analysis used summary-level data, stratified analyses were not possible. Fourth, the conceptual DAG was expanded to include clinically important EP risk factors in addition to smoking and alcohol consumption, namely pelvic inflammatory disease or other pelvic/tubal infection, previous ectopic pregnancy, prior pelvic or tubal surgery or injury, intrauterine device use, and conception by assisted reproductive technology. These factors were considered in the conceptual confounding framework; however, the PhenoScanner database did not provide complete GWAS coverage for all of these clinical phenotypes. Therefore, the absence of a cataloged association was not interpreted as evidence that a retained instrument was entirely free from confounding or horizontal pleiotropy. Fifth, the ectopic-pregnancy outcome was defined using hospital discharge and cause-of-death registry codes rather than individual clinical adjudication. Because formal endpoint-specific validation metrics and individual imaging, surgical, pathology, or chart-review confirmation were not available in the public FinnGen documentation, outcome misclassification, including potential variation in diagnostic certainty or ectopic-pregnancy subtype, cannot be fully excluded. Future studies, including well-designed clinical studies, are needed to examine the potential role of vitamin D and related pathways in the prevention and treatment of EP.

## 6. Conclusion

In this MR study of European-ancestry participants, genetically predicted higher circulating 25(OH)D levels were associated with a higher risk of registry-defined EP. These findings should not be interpreted as establishing serum 25(OH)D as a diagnostic marker for EP. Rather, vitamin D may represent a potential associated risk factor that, pending confirmation in clinical studies, could be considered only alongside established clinical signs and symptoms of EP. Further clinical and mechanistic studies are required before any diagnostic or clinical risk-stratification application can be considered.

## Acknowledgments

Data in the European population on uterine 25-hydroxyvitamin D levels and ectopic pregnancy are available through the GWAS database. The authors would like to thank all these researchers for their selless sharing.

## Author contributions

**Conceptualization:** Pei-Jin Zhang, Xiao-Dong Fan.

**Data curation:** Pei-Jin Zhang, Shao Bo, Shuang Liang.

**Formal analysis:** Pei-Jin Zhang, Shao Bo, Shuang Liang, Hong-Yuan Zhang.

**Funding acquisition:** Xiao-Dong Fan.

**Investigation:** Pei-Jin Zhang, Shao Bo, Shuang Liang.

**Methodology:** Pei-Jin Zhang, Shao Bo, Shuang Liang, Hong-Yuan Zhang.

**Project administration:** Pei-Jin Zhang.

**Resources:** Hong-Yuan Zhang.

**Software:** Pei-Jin Zhang, Shao Bo, Shuang Liang, Hong-Yuan Zhang.

**Supervision:** Shao Bo, Xiao-Dong Fan.

**Validation:** Xiao-Dong Fan.

**Visualization:** Shuang Liang.

**Writing – original draft:** Pei-Jin Zhang, Shao Bo.

**Writing – review & editing:** Xiao-Dong Fan.




