# Supplementary material for: The causal associations between 25(OH) vitamin D levels and ectopic pregnancy: A 2-sample Mendelian randomization study
Source: Medicine (Baltimore). 2026 Sep 18;105(38):e50657. doi: 10.1097/MD.0000000000050657 (PMC13593265; doi:10.1097/MD.0000000000050657)
Supplement: Supplementary file 1 [file medi-105-e50657-s001.docx]

| **Supplementary Table S1：Source of exposure genome-wide association study summary data** | | | | |  |
| --- | --- | --- | --- | --- | --- |
| Trait | Ancestry | Number of participants | Number of SNPs used | Web source |  |
|  |  |  |  |  |  |
| serum 25(OH)D | European | 496,946 | 103 | <https://www.frontiersin.org/articles/10.3389/fnut.2023.1111950/full#supplementary-material> |  |
| circulating 25(OH)D | European | 441,291 | 16,668,957 | <https://gwas.mrcieu.ac.uk/datasets/ieu-b-4808> |  |
| ectopic pregnancy | European | 3,111/89,340 | 16,378,923 | https://gwas.mrcieu.ac.uk/datasets/finn-b-O15_PREG_ECTOP |  |
| ever smoked | European | 461,066 | 9,851,867 | <https://gwas.mrcieu.ac.uk/datasets/ukb-b-20261> |  |
| alcohol intake | European | 428,117 | 9,851,867 | <https://gwas.mrcieu.ac.uk/datasets/ukb-b-3460> |  |
