# Supplementary material for: The causal associations between 25(OH) vitamin D levels and ectopic pregnancy: A 2-sample Mendelian randomization study
Source: Medicine (Baltimore). 2026 Sep 18;105(38):e50657. doi: 10.1097/MD.0000000000050657 (PMC13593265; doi:10.1097/MD.0000000000050657)
Supplement: Supplementary file 2 [file medi-105-e50657-s002.docx]

| **Supplementary Table S2： SNPs were included to establish the genetic IVs for serum 25(OH)D levels** | | | | | | | | | |
| --- | --- | --- | --- | --- | --- | --- | --- | --- | --- |
| SNP | effect_allele | other_allele | beta | eaf | chr | pos | se | pval |  |
| rs10277163 | G | A | -0.0143336 | 0.254715 | 7 | 21569089 | 0.0023507 | 1.08E-09 |  |
| rs1038165 | T | C | 0.0115149 | 0.579451 | 12 | 68665940 | 0.00205629 | 2.15E-08 |  |
| rs1042034 | T | C | -0.0151254 | 0.792138 | 2 | 21225281 | 0.00250017 | 1.45E-09 |  |
| rs10438978 | C | T | -0.0172243 | 0.820277 | 18 | 47158186 | 0.00264437 | 7.34E-11 |  |
| rs1047891 | A | C | -0.0133984 | 0.317078 | 2 | 211540507 | 0.00218016 | 7.96E-10 |  |
| rs1048328 | A | G | 0.0313497 | 0.0797967 | 19 | 51527364 | 0.00374376 | 5.58E-17 |  |
| rs10859995 | C | T | -0.0436264 | 0.579841 | 12 | 96375682 | 0.00205452 | 4.60E-100 |  |
| rs11023159 | C | T | 0.0482117 | 0.0325143 | 11 | 14262063 | 0.00572507 | 3.73E-17 |  |
| rs11076175 | G | A | 0.0229033 | 0.175666 | 16 | 57006378 | 0.00266997 | 9.64E-18 |  |
| rs111515741 | A | G | -0.0487364 | 0.0173081 | 11 | 14370944 | 0.00779044 | 3.95E-10 |  |
| rs11207969 | G | A | 0.0209396 | 0.351365 | 1 | 62911751 | 0.00212672 | 7.14E-23 |  |
| rs11264361 | G | T | 0.0174875 | 0.251408 | 1 | 155289545 | 0.00234081 | 7.97E-14 |  |
| rs1128535 | T | C | 0.0164131 | 0.500049 | 3 | 49866392 | 0.0020293 | 6.06E-16 |  |
| rs115288876 | A | G | 0.0788065 | 0.0433211 | 1 | 152000117 | 0.00498229 | 2.36E-56 |  |
| rs11542462 | A | G | -0.0247803 | 0.133511 | 16 | 82033810 | 0.00298265 | 9.72E-17 |  |
| rs11600054 | A | G | 0.0681747 | 0.0100965 | 11 | 14690511 | 0.0101478 | 1.84E-11 |  |
| rs117300835 | A | G | -0.334985 | 0.0133061 | 11 | 15118975 | 0.00886005 | 1.00E-200 |  |
| rs11791258 | A | G | 0.0140808 | 0.191072 | 9 | 107632644 | 0.00258052 | 4.85E-08 |  |
| rs11867297 | T | C | 0.0135432 | 0.38533 | 17 | 66433493 | 0.00209454 | 1.01E-10 |  |
| rs12056768 | G | T | -0.0231961 | 0.584044 | 8 | 116988527 | 0.00206406 | 2.65E-29 |  |
| rs12153819 | T | C | -0.0178178 | 0.123154 | 6 | 83824703 | 0.0030906 | 8.16E-09 |  |
| rs12283049 | G | A | -0.0564566 | 0.234433 | 11 | 14690192 | 0.00240614 | 9.62E-122 |  |
| rs12324720 | A | G | -0.0149159 | 0.174629 | 15 | 64092140 | 0.00267451 | 2.45E-08 |  |
| rs12462826 | A | G | -0.0132119 | 0.369566 | 19 | 11955767 | 0.0021149 | 4.18E-10 |  |
| rs12775091 | T | C | 0.0155618 | 0.213441 | 10 | 91524012 | 0.00247693 | 3.33E-10 |  |
| rs13076508 | C | T | 0.0250544 | 0.0535034 | 3 | 52407805 | 0.00451036 | 2.78E-08 |  |
| rs1321247 | T | A | -0.0221847 | 0.101578 | 6 | 25662873 | 0.00336578 | 4.36E-11 |  |
| rs13294734 | T | C | 0.012568 | 0.466128 | 9 | 80710910 | 0.00205837 | 1.02E-09 |  |
| rs1343776 | A | G | 0.0180762 | 0.22133 | 1 | 41757718 | 0.00245028 | 1.62E-13 |  |
| rs1384687 | A | G | -0.0168656 | 0.132271 | 8 | 61525963 | 0.00299675 | 1.82E-08 |  |
| rs142004400 | C | A | -0.0310034 | 0.0342066 | 14 | 50829560 | 0.00559561 | 3.01E-08 |  |
| rs142158911 | A | G | 0.026284 | 0.111826 | 19 | 11190534 | 0.00323446 | 4.43E-16 |  |
| rs144965707 | A | G | -0.0348143 | 0.0618296 | 11 | 14093639 | 0.00421754 | 1.52E-16 |  |
| rs1532085 | G | A | 0.0252805 | 0.616672 | 15 | 58683366 | 0.00208639 | 8.60E-34 |  |
| rs1627043 | C | G | -0.0486441 | 0.0332414 | 11 | 71110175 | 0.00566106 | 8.49E-18 |  |
| rs1684600 | T | C | -0.0125301 | 0.298695 | 16 | 4594671 | 0.00221719 | 1.59E-08 |  |
| rs17207784 | C | T | -0.0134939 | 0.324195 | 6 | 22768668 | 0.0021713 | 5.14E-10 |  |
| rs17473257 | A | G | -0.0611372 | 0.0172466 | 11 | 14283186 | 0.00780044 | 4.59E-15 |  |
| rs1800588 | T | C | -0.0305021 | 0.214964 | 15 | 58723675 | 0.00246932 | 4.73E-35 |  |
| rs1841850 | C | A | 0.0304395 | 0.117085 | 20 | 52718555 | 0.00316491 | 6.73E-22 |  |
| rs1858889 | C | A | 0.0134514 | 0.502532 | 7 | 107117447 | 0.00203065 | 3.49E-11 |  |
| rs1949633 | C | T | 0.0114162 | 0.605759 | 3 | 153758806 | 0.00208637 | 4.45E-08 |  |
| rs2037511 | A | G | 0.0176624 | 0.165924 | 18 | 61366207 | 0.00272732 | 9.41E-11 |  |
| rs2229742 | C | G | -0.0249837 | 0.104659 | 21 | 16339172 | 0.00331409 | 4.75E-14 |  |
| rs2245133 | C | T | -0.0212917 | 0.164339 | 6 | 131931092 | 0.00273999 | 7.80E-15 |  |
| rs2297991 | C | T | 0.0127547 | 0.718478 | 10 | 113913222 | 0.00225578 | 1.57E-08 |  |
| rs2398113 | G | A | -0.0117606 | 0.423526 | 10 | 10078742 | 0.00205797 | 1.10E-08 |  |
| rs2494429 | G | A | -0.0148459 | 0.822999 | 1 | 2339395 | 0.00267333 | 2.80E-08 |  |
| rs2595644 | T | G | -0.0122625 | 0.384889 | 7 | 43980540 | 0.00209685 | 4.97E-09 |  |
| rs2710651 | A | G | -0.0115892 | 0.526101 | 2 | 63166379 | 0.00203474 | 1.23E-08 |  |
| rs2756119 | A | G | 0.0121434 | 0.38143 | 14 | 104001517 | 0.0021104 | 8.71E-09 |  |
| rs2807834 | G | T | -0.0150625 | 0.68514 | 1 | 220970593 | 0.00218678 | 5.66E-12 |  |
| rs2847500 | A | G | -0.022548 | 0.123204 | 11 | 120114421 | 0.00308665 | 2.77E-13 |  |
| rs290400 | A | G | -0.0130967 | 0.665472 | 20 | 52698179 | 0.00216338 | 1.41E-09 |  |
| rs325393 | T | G | -0.0136497 | 0.278218 | 15 | 100229260 | 0.00227676 | 2.03E-09 |  |
| rs34186890 | G | A | -0.0156853 | 0.259637 | 3 | 141720712 | 0.00231839 | 1.33E-11 |  |
| rs34726834 | T | C | 0.014013 | 0.252209 | 8 | 25889606 | 0.00234858 | 2.42E-09 |  |
| rs35270497 | T | C | 0.0156723 | 0.176236 | 2 | 38259872 | 0.0026815 | 5.08E-09 |  |
| rs35823191 | C | T | -0.0232636 | 0.342049 | 1 | 17560123 | 0.00214072 | 1.65E-27 |  |
| rs3732220 | A | G | -0.0478406 | 0.0852646 | 2 | 234627048 | 0.00363266 | 1.31E-39 |  |
| rs3829251 | A | G | -0.114453 | 0.133277 | 11 | 71194559 | 0.00298056 | 1.00E-200 |  |
| rs4147536 | C | A | -0.0148038 | 0.788613 | 4 | 100239112 | 0.00249007 | 2.76E-09 |  |
| rs4348160 | G | T | -0.0258401 | 0.326968 | 4 | 70017531 | 0.00216263 | 6.62E-33 |  |
| rs4364259 | A | G | 0.0172408 | 0.198665 | 4 | 15892159 | 0.00256676 | 1.86E-11 |  |
| rs4580037 | C | A | -0.0135627 | 0.285578 | 13 | 55702646 | 0.00225079 | 1.68E-09 |  |
| rs512083 | C | T | 0.0122172 | 0.462488 | 1 | 46027355 | 0.00204286 | 2.23E-09 |  |
| rs57601828 | T | A | 0.011542 | 0.394508 | 12 | 93204367 | 0.00208192 | 2.96E-08 |  |
| rs5770794 | T | C | -0.0133141 | 0.3143 | 22 | 50880781 | 0.00221143 | 1.74E-09 |  |
| rs6129648 | G | A | 0.0140631 | 0.379837 | 20 | 39231118 | 0.00210628 | 2.44E-11 |  |
| rs61698755 | C | T | -0.011465 | 0.560041 | 17 | 79257880 | 0.0020505 | 2.25E-08 |  |
| rs61747728 | T | C | 0.0303061 | 0.0385611 | 1 | 179526214 | 0.00526894 | 8.83E-09 |  |
| rs61813875 | G | C | 0.0821291 | 0.0248209 | 1 | 152536650 | 0.00658876 | 1.16E-35 |  |
| rs61887421 | C | T | -0.036726 | 0.0300871 | 11 | 70949673 | 0.00597754 | 8.05E-10 |  |
| rs635634 | T | C | -0.0150476 | 0.186573 | 9 | 136155000 | 0.0026042 | 7.55E-09 |  |
| rs6438900 | G | C | 0.0150486 | 0.256034 | 3 | 125148287 | 0.00234031 | 1.27E-10 |  |
| rs6834488 | T | C | -0.01445 | 0.422833 | 4 | 88178919 | 0.00205911 | 2.26E-12 |  |
| rs71599974 | G | A | 0.0257378 | 0.148048 | 4 | 71765339 | 0.00286182 | 2.39E-19 |  |
| rs727857 | A | G | -0.0120548 | 0.61181 | 2 | 58981967 | 0.00209882 | 9.27E-09 |  |
| rs733454 | T | C | 0.0188545 | 0.0991663 | 11 | 76477721 | 0.00340001 | 2.93E-08 |  |
| rs73413596 | C | T | 0.0223468 | 0.0740317 | 12 | 111582630 | 0.00388928 | 9.15E-09 |  |
| rs742493 | C | T | 0.0183528 | 0.112871 | 6 | 40998167 | 0.00320607 | 1.04E-08 |  |
| rs7528419 | G | A | 0.0215389 | 0.22441 | 1 | 109817192 | 0.00243165 | 8.17E-19 |  |
| rs7569755 | A | G | 0.0136395 | 0.288618 | 2 | 118648261 | 0.00225626 | 1.49E-09 |  |
| rs7580771 | T | G | -0.0165625 | 0.175983 | 2 | 101428119 | 0.0026652 | 5.15E-10 |  |
| rs7712001 | G | T | 0.011939 | 0.440176 | 5 | 148020950 | 0.00206215 | 7.05E-09 |  |
| rs77532868 | T | C | 0.0259557 | 0.0521528 | 10 | 88081438 | 0.00456301 | 1.28E-08 |  |
| rs77924615 | A | G | -0.0152475 | 0.194374 | 16 | 20392332 | 0.00259019 | 3.94E-09 |  |
| rs77960347 | G | A | -0.0525688 | 0.0127013 | 18 | 47109955 | 0.00905963 | 6.53E-09 |  |
| rs78649910 | A | T | -0.0191377 | 0.105728 | 4 | 3482213 | 0.00332162 | 8.33E-09 |  |
| rs7955128 | T | A | 0.0130617 | 0.52053 | 12 | 38684121 | 0.00203856 | 1.48E-10 |  |
| rs804281 | G | A | 0.015895 | 0.583498 | 8 | 11611865 | 0.00205997 | 1.20E-14 |  |
| rs8107974 | T | A | 0.0355672 | 0.0763013 | 19 | 19388500 | 0.00382316 | 1.36E-20 |  |
| rs8121940 | G | C | -0.0435612 | 0.19762 | 20 | 52742306 | 0.00254898 | 1.77E-65 |  |
| rs9375037 | C | A | 0.0117059 | 0.443168 | 6 | 121856794 | 0.00205423 | 1.21E-08 |  |
| rs964184 | C | G | 0.0406845 | 0.867234 | 11 | 116648917 | 0.00298936 | 3.50E-42 |  |
| rs9847248 | A | G | -0.0123077 | 0.712969 | 3 | 18804655 | 0.00224484 | 4.19E-08 |  |
| rs986649 | G | A | 0.0128637 | 0.321628 | 5 | 118668050 | 0.00217811 | 3.51E-09 |  |
| rs9946771 | T | C | -0.0233992 | 0.0663426 | 18 | 28918628 | 0.00407655 | 9.47E-09 |  |
